# Application of ray-traced tropospheric slant delays to geodetic VLBI analysis

**DOI:** 10.1007/s00190-017-1000-7

**Published:** 2017-02-22

**Authors:** Armin Hofmeister, Johannes Böhm

**Affiliations:** Department of Geodesy and Geoinformation, Technische Universität Wien, Gußhausstraße 27-29, 1040 Vienna, Austria

**Keywords:** Atmospheric effects, Tropospheric delays, Ray-tracing, VLBI, Numerical weather model

## Abstract

The correction of tropospheric influences via so-called path delays is critical for the analysis of observations from space geodetic techniques like the very long baseline interferometry (VLBI). In standard VLBI analysis, the a priori slant path delays are determined using the concept of zenith delays, mapping functions and gradients. The a priori use of ray-traced delays, i.e., tropospheric slant path delays determined with the technique of ray-tracing through the meteorological data of numerical weather models (NWM), serves as an alternative way of correcting the influences of the troposphere on the VLBI observations within the analysis. In the presented research, the application of ray-traced delays to the VLBI analysis of sessions in a time span of 16.5 years is investigated. Ray-traced delays have been determined with program RADIATE (see Hofmeister in Ph.D. thesis, Department of Geodesy and Geophysics, Faculty of Mathematics and Geoinformation, Technische Universität Wien. http://resolver.obvsg.at/urn:nbn:at:at-ubtuw:1-3444, 2016) utilizing meteorological data provided by NWM of the European Centre for Medium-Range Weather Forecasts (ECMWF). In comparison with a standard VLBI analysis, which includes the tropospheric gradient estimation, the application of the ray-traced delays to an analysis, which uses the same parameterization except for the a priori slant path delay handling and the used wet mapping factors for the zenith wet delay (ZWD) estimation, improves the baseline length repeatability (BLR) at 55.9% of the baselines at sub-mm level. If no tropospheric gradients are estimated within the compared analyses, 90.6% of all baselines benefit from the application of the ray-traced delays, which leads to an average improvement of the BLR of 1 mm. The effects of the ray-traced delays on the terrestrial reference frame are also investigated. A separate assessment of the RADIATE ray-traced delays is carried out by comparison to the ray-traced delays from the National Aeronautics and Space Administration Goddard Space Flight Center (NASA GSFC) (Eriksson and MacMillan in http://lacerta.gsfc.nasa.gov/tropodelays, 2016) with respect to the analysis performances in terms of BLR results. If tropospheric gradient estimation is included in the analysis, 51.3% of the baselines benefit from the RADIATE ray-traced delays at sub-mm difference level. If no tropospheric gradients are estimated within the analysis, the RADIATE ray-traced delays deliver a better BLR at 63% of the baselines compared to the NASA GSFC ray-traced delays.

## Introduction

The influence of the troposphere is one of the major error sources on observations of space geodetic methods like the very long baseline interferometry (VLBI) or the global navigation satellite systems (GNSS). While traveling through the atmosphere the observational signal is affected by the atmosphere in terms of the propagation path and the propagation speed. The ionospheric influence on the signal can be corrected by utilizing the dispersion effect through observing at two different signal frequencies (Nilsson et al. 2013).

In common VLBI analysis, the tropospheric effect is eliminated from the observations in form of so-called slant path delays since the effects on the path and propagation speed can be expressed as time delays. The determination of the tropospheric delays is usually done using zenith delays and mapping functions like the Vienna mapping functions 1 (VMF1; see Böhm et al. 2006). Commonly, the zenith hydrostatic delay determined from Saastamoinen’s equation (see Saastamoinen 1972) with the surface pressure at the station is used to derive the a priori slant delay with the utilization of a mapping function. Within the subsequent analysis, zenith wet delays (ZWD) are estimated and used together with the zenith hydrostatic delays (ZHD) and the mapping functions for the final tropospheric delay estimation and the correction of the observations.

An alternative approach compared to the model, i.e., mapping function, based a priori slant delay determination, is the utilization of the ray-tracing technique for the reconstruction of the true signal path and the subsequent delay calculation along the path. Different authors have investigated the ray-tracing technique for tropospheric delay estimation in the past. Important research and related studies can be found in Hobiger et al. (2008); Nafisi et al. (2012a, b); Eriksson et al. (2014).

The ray-tracing method usually uses meteorological data directly from numerical weather models (NWM) for both the path and the delay determination, which means that a very accurate data source is utilized for the complete processing and there is no need to rely on any other model data except for supplemental standard atmosphere model data. Thus, the so-called ray-traced delays are a promising way to improve the VLBI analysis. The presented research therefore focuses on the application of ray-traced delays to the VLBI analysis. It is investigated in comparison to common VLBI analyses if the solutions benefit from applying ray-traced delays to the analyses in terms of baseline length repeatability (BLR). Furthermore, the impacts on the terrestrial reference frame (TRF) are determined. The research is carried out by using ray-traced delays determined with program RADIATE (see Hofmeister 2016). Also an assessment of the RADIATE ray-traced delays is presented by investigating the analysis performance in comparison with applying the ray-traced delays from the National Aeronautics and Space Administration Goddard Space Flight Center (NASA GSFC; Eriksson and MacMillan 2016).

## Ray-traced tropospheric slant delays

The approach of ray-tracing is utilized in many different scientific fields and can also be applied for the tropospheric slant path delay retrieval of VLBI observations.

### Theoretical background

In order to derive the slant path delays for geodetic VLBI observations the true signal path is determined via ray-tracing. For this task, it is necessary to consider the physics behind the propagation of the signals in the atmosphere, i.e., of microwave signals as in case of VLBI.

Maxwell’s equations describe the propagation of electromagnetic waves (Jackson 1998). From these, it is possible to derive the main equation of ray-tracing, the Eikonal equation (Nilsson et al. 2013). The Eikonal equation itself is the solution of the Helmholtz equation in the case of electromagnetic wave propagation in a slowly varying medium with negligible diffraction effects (Iizuka 2008; Wheelon 2001; Born and Wolf 1999). This means that the geometrical optics approximation is applied, which enables the description of the signal propagation as a ray. Assuming that the propagation medium is free of currents and charges and that the wavelength is small, the Eikonal equation can be written as (Born and Wolf 1999; Nilsson et al. 2013)

1 $$\begin{aligned} ||\nabla L ||^2 = n(\mathbf {r})^2 \end{aligned}$$

with $\nabla L$ representing the different components of the ray. *L* describes the optical path length and *n*, which is dependent on the position $\mathbf {r}$ along the ray, describes the refractive index of the medium. By $L(\mathbf {r}) = const.$ so-called geometric wave surfaces or geometrical wave fronts are derived (Born and Wolf 1999; Nilsson et al. 2013).

Solving the Eikonal equation is the strict method for determining the ray path and thus also for deriving the tropospheric delay. The Eikonal equation is used to describe the three-dimensional (3D) propagation of a ray path through the atmosphere. Since the solution is based on six partial differential equations, the calculations are very time consuming in case of operational ray-tracing application. Thus, it is an adequate step to simplify the strict ray propagation theory for practical implementations with large data sets. A reasonable first approximation is the restriction of the ray to a two-dimensional (2D) path solution by fixing the azimuth angle of the observation and thus prohibiting any ray propagations out of the vertical plane. The received 2D ray-tracing method can be further simplified. Depending on the introduced approximations different realizations of the strict ray-tracing can be found. An example of a simple but fast 2D approach is the piecewise-linear (PWL) method, which approximates the ray path by introducing PWL ray segments along the ray.

Investigations on the accuracy of different practical implementations of the ray-tracing method have been carried out by Hobiger et al. (2008); Nafisi et al. (2012a); Hofmeister (2016).

The ray-traced delays used in the presented research have been determined with the ray-tracing program RADIATE utilizing the implemented PWL ray-tracing approach. Please refer to Hofmeister (2016) for more information on program RADIATE, the inherited ray-tracing methods and how they are implemented.

From Eq. (1) it is obvious that the knowledge about the refractive index *n* is needed in order to determine the signal path. In practice, it is more convenient to use the refractivity, which can be determined as

2 $$\begin{aligned} N = (n-1) \cdot 10^6. \end{aligned}$$

The refractivity *N*, valid at radio frequencies, can be determined according to Smith and Weintraub (1953) as

3 $$\begin{aligned} N = k_1 \frac{p_\mathrm{d}}{T} + k_2 \frac{p_\mathrm{w}}{T} + k_3 \frac{p_\mathrm{w}}{T^2}, \end{aligned}$$

where $p_\mathrm{d}$ is the partial pressure of dry air in hPa, $p_\mathrm{w}$ is the partial pressure of water vapor in hPa and *T* is the temperature in degrees K. The variables $k_1$, $k_2$ and $k_3$ denote the so-called refractivity coefficients, which have been determined in numerous different laboratory measurements (Nilsson et al. 2013). For program RADIATE, and thus for the studies presented here the “best average” solution of Rüeger (2002a, b) is used as shown in Table 1.

**Table 1:** “Best average” refractivity coefficients according to Rüeger (2002a, b)

$k_1$	77.6890 [K/hPa]
$k_2$	71.2952 [K/hPa]
$k_3$	375463 [K$^2$/hPa]

By using meteorological data provided by NWM the refractivity can be calculated.

From the VLBI observation the outgoing (or vacuum) elevation angle is known. This is the angle at which the signal from the radio source enters the neutral atmosphere. Since the ray-tracing approach needs the station elevation angle as starting point, the signal path has to be determined iteratively by subsequent adaptation of an initial elevation angle setting at the station position in order to reconstruct the observed outgoing elevation angle and thus the true signal path of the observation through the atmosphere.

Following the path retrieval the delay along the derived path can be calculated. For this task again the refractivities are needed. Applying the geometrical optics approximation for the delay determination, it is sufficient to know the refractivities along the path (Nilsson et al. 2013). The electric path length *L*, i.e., the travel time of a signal propagating through the atmosphere along the ray *S*, is determined as (Nilsson et al. 2013)

4 $$\begin{aligned} L = \int _S n(s) {{\mathrm{d}}}s. \end{aligned}$$

According to Nilsson et al. (2013) the atmospheric delay $\Delta L$ can be described as the excess in the electric path length of a signal due to the atmospheric influences compared to the vacuum signal propagation and it can be determined as

5 $$\begin{aligned} \Delta L= & {} L - G \nonumber \\= & {} \int _S n(s) {{\mathrm{d}}}s - G \nonumber \\= & {} 10^{-6} \int _S N_\mathrm{h}(s) {{\mathrm{d}}}s + 10^{-6} \int _S N_\mathrm{w}(s) {{\mathrm{d}}}s + S - G \nonumber \\= & {} \Delta L_\mathrm{h} + \Delta L_\mathrm{w} + S - G, \end{aligned}$$

where *G* is the straight line path length along which the signal would propagate in vacuum and *S* is the true signal path. Due to the effects of reduced signal propagation speed, a delay is introduced, which can be split into the hydrostatic and wet delays $\Delta L_\mathrm{h}$ and $\Delta L_\mathrm{w}$ derived from the hydrostatic and wet refractivities $N_\mathrm{h}$ and $N_\mathrm{w}$ accounting for the hydrostatic and wet or more accurately the non-hydrostatic effects of the atmosphere. Furthermore, the signal path does not follow the straight line *G*, and thus a so-called geometric bending effect $g_\mathrm{bend}=S-G$ is additionally part of the total atmospheric delay $\Delta L$.

Further or more detailed information on the ray-tracing and the delay retrieval using different ray-tracing approaches can be found in Hofmeister (2016).

## Application of ray-traced delays to the VLBI analysis

In the following, a detailed research on the impact of the application of ray-traced delays to the VLBI analysis is presented. Based on ray-traced delays from program RADIATE (see Hofmeister 2016), their influence on the BLR as well as on the TRF is determined.

### Data for the research

The choice of appropriate data is of fundamental concern for every research. Thus, the observational data set, which is processed in the VLBI analysis as well as for which ray-traced delays are determined, has been selected to keep the most reliable VLBI data sessions.

#### VLBI observational data

The chosen observational data set contains initially 2461 VLBI sessions between January 1999 and June 2015. This interval will be called 1999.0–2015.5 in the following. Only reliable sessions with a sufficiently high number of participating stations have been selected. Thus, no so-called intensive sessions are part of the data set since they only involve two to three stations. The time span of 16.5 years of VLBI observations ensures that generally valid conclusions can be drawn from the results.

In order to ensure reliable comparisons of the BLR results and TRF solutions, 121 sessions are excluded from the subsequent comparisons of the analysis results since in at least one of the carried out analyses their solution is either close to singular, has an a posteriori standard deviation of unit weight^1^ of larger than 3 or contains at least one baseline length estimate with a formal error of more than 10 cm. Thus, the following comparisons in this chapter contain and use the results of 2340 reliable sessions.

#### Ray-traced tropospheric slant delays

For the above-defined observational data set, the corresponding ray-traced tropospheric slant delays are determined with program RADIATE.

The meteorological data needed for the determination of the refractivities, which are required for the path and delay retrieval, are taken from the NWM of the European Centre for Medium-Range Weather Forecasts (ECMWF). For observations until 31.12.2007 the ECMWF Re-Analysis-Interim (ERA-Interim) NWM is used, for later observation times the ECMWF operational NWM is utilized.^2^ The chosen horizontal resolution^3^ of the NWM data is $1{^\circ } \times 1{^\circ }$ and in the vertical 25 total pressure levels are used. The vertical resolution of the meteorological data is significantly enhanced by interpolation at discrete height levels and extended up to 84 km by the use of the US Standard Atmosphere 1976 (see COESA 1976) for the vertical interpolation. The temporal resolution of the used NWM data is 6 h, i.e., four NWM epochs per day are utilized. For each observation, two ray-traced delays are determined. One at each of the two NWM epochs, which surround the observation time. These are linearly interpolated to receive a final delay that is valid at the exact observation time. For the ray-tracing itself, the PWL approach is utilized. More detailed information on the operational ray-tracing settings of program RADIATE as well as investigations for the finding of these most suitable settings can be found in Hofmeister (2016). Hofmeister (2016) and this article serve as references if ray-traced delays and/or the corresponding products of program RADIATE are used.

### VLBI analysis parameterization

The analysis of the VLBI sessions is carried out with the Vienna VLBI Software (VieVS; see Böhm et al. 2012). For the investigations of the impact of the ray-traced delays on the VLBI analysis, four different parameterizations are considered:

1. $${\textit{VieVS}}$$
  A typical VLBI analysis parameterization.
  Estimation of tropospheric gradients is included.
2. $${\textit{VieVS}\, \textit{no} \,\textit{gradients}}$$
  In principle identical to parameterization 1, but:
  No estimation of tropospheric gradients.
3. $${\textit{RADIATE}}$$
  A typical VLBI analysis parameterization identical to parameterization 1 with estimation of tropospheric gradients, but:
  Application of ray-traced delays as a priori slant delays to the VLBI analysis.
  The wet mapping factors from the ray-tracing results are utilized for the ZWD estimation instead of those from the VMF1.
4. $${\textit{RADIATE}\, \textit{no} \,\textit{gradients}}$$
  In principle identical to parameterization 3, but:
  No estimation of tropospheric gradients.
  The difference to parameterization 2 is the application of ray-traced delays as a priori slant delays to the VLBI analysis and the use of the wet mapping factors from the ray-tracing results instead of those from the VMF1 for the ZWD estimation.

The Tables 2 and 3 describe the most important settings for the a priori models and the parameter estimation, which are common to all parameterizations used in this work.

**Table 2:** Important a priori model settings common to all parameterizations in this work

Option	Setting
Reference frames	
TRF	VieVS-internal TRF solution
Celestial Reference Frame (CRF)	International Celestial Reference Frame 2nd realization (ICRF2) (see Fey et al. 2015)
Ephemerides	JPL 421 model (see Folkner et al. 2009) from the National Aeronautics and Space Administration Jet Propulsion Laboratory (NASA JPL)
Station correction models	
Solid Earth tides	Following the guidelines of the IERS Conventions 2010 (see Petit and Luzum 2010). IERS denotes the International Earth Rotation and Reference Systems Service
Tidal ocean loading	FES2004 (see Lyard et al. 2006)
Tidal atmosphere loading	Atmospheric Pressure Loading (APL) model of Technische Universität Wien called Vienna-APL (see Wijaya et al. 2013)
Non-tidal atmosphere loading	Vienna-APL model (see Wijaya et al. 2013)
Pole tide	Following the guidelines of the IERS Conventions 2010 (see Petit and Luzum 2010)
Ocean pole tide	Following the guidelines of the IERS Conventions 2010 (see Petit and Luzum 2010)
Mean pole model	Cubic model following the guidelines of the IERS Conventions 2010 (see Petit and Luzum 2010)
Thermal antenna deformation	According to Nothnagel (2009)
Earth Orientation Parameters (EOP)	A priori time series IERS 08 C04. Application according to the IERS Conventions 2010 (see Petit and Luzum 2010)

**Table 3:** Parameter estimation settings common to all parameterizations in this work

Parameter	Setting
Clock	PWL offsets + rate + quadratic term, interval of 60 min., relative constraints of 1.3 cm after 60 min. Estimations are per clock and with respect to a reference clock
ZWD	PWL offsets, interval of 30 min., relative constraints of 1.5 cm after 30 min. The wet mapping factors are used as partial derivatives for the estimation of the ZWD. Depending on not applying or applying the ray-traced delays to the analysis, the utilized wet mapping factors originate either from the VMF1 or from the ray-tracing results
EOP	Offsets estimated session-wise
Station coordinates	Offsets estimated session-wise. No-Net-Translation (NNT) and No-Net-Rotation (NNR) conditions
Source coordinates	Estimation only of sources not provided by the ICRF2. Offsets estimated session-wise

Supplementary to these definitions, Table 4 shows the special settings and thus the differences between the parameterizations 1–4 used for the assessment of the analysis impact of the ray-traced delays.

**Table 4:** Special settings and thus differences between the individual analysis parameterizations 1–4

Parameterization	1	2	3	4
A priori tropospheric delay				
ZHD from Saastamoinen’s equation (see Saastamoinen 1972) mapped with VMF1 (see Böhm et al. 2006) to the elevation angle of the observation and then used as a priori tropospheric delay	$\checkmark$	$\checkmark$		
Ray-traced slant total delay utilized as a priori tropospheric delay			$\checkmark$	$\checkmark$
Tropospheric gradient model and estimation settings				
No a priori gradient model introduced, but North- and East-gradients estimated according to the model of Chen and Herring (1997) as PWL offsets in the interval of 120 min. with relative constraints of 0.05 cm after 120 min	$\checkmark$		$\checkmark$	
No a priori gradient model introduced and no gradients estimated		$\checkmark$		$\checkmark$

In order to determine the influence of the ray-traced delays on the VLBI analysis, the results from the different parameterizations are compared pairwise. The analysis results from parameterization 1 are compared to those from parameterization 3 to see the effect in terms of a typical VLBI analysis. The comparison of the results from parameterization 2 to those from parameterization 4 is used to assess the value of the ray-traced delays if no tropospheric gradients are estimated within the VLBI analysis.

The impact assessments are done with respect to the BLR and the TRF solutions.

### Influence of ray-traced delays on the baseline length repeatability

The BLR serves as an important assessment parameter for the validation of the accuracy of a VLBI analysis. Therefore, the BLR is determined in this research for each of the analysis solutions from the different parameterizations.

Basically the BLR is calculated as the unbiased weighted standard deviation of the session-wise estimates of a specific baseline length between two stations. Since the time span covered by the set of analyzed sessions is long, trends have to be subtracted from the baseline length estimates in order to receive comparable estimates. The trends result from plate tectonics or station discontinuities due to earthquakes or relocations and thus may change with time depending on the occurrence of such break events. Therefore, a separate linear trend function is determined for each break interval in the time series of a specific baseline and corresponding trends are subtracted from the baseline length estimates. Then a separate BLR value is determined for each specific break interval of the baseline in form of the unbiased weighted standard deviation of the trend-reduced length estimates. The weights for the estimation of the BLR are the inverses of the squared formal errors of the baseline length estimates, which are determined from the formal coordinate errors of the baseline forming stations using the corresponding covariances. The final BLR result of a specific baseline is determined by calculating the weighted mean of the single BLR values from the separate break intervals. Here the weights are the numbers of baseline length estimates, which have been used to determine the BLR values of the individual break intervals. In order to be able to accurately determine the trend function for each break interval, a minimum number of 30 baseline length estimates in a specific break interval must be available to avoid unreliable trend values, which would be reduced from the baseline length estimates to determine the BLR for the break interval. If this minimum limit of baseline length estimates is not reached, no BLR is calculated for this interval.

In order to compare the BLR results and thus assess the impact of the ray-traced delays on the VLBI analysis, differences in the BLR ($\Delta \hbox {BLR}$) are computed between the solutions from two different analysis parameterizations. One solution is derived from an analysis without applying ray-traced delays and the other solution is derived from using ray-traced delays. According to the formalism of the computed differences, positive $\Delta \hbox {BLR}$ denote that the BLR is improved if the ray-traced delays are applied to the analysis.

Also the relative amount of change of the BLR ($\delta \hbox {BLR}$) is assessed. It is given by

6 $$\begin{aligned} \delta \hbox {BLR} [\%] = \frac{\hbox {BLR}_\mathrm{Param.~1} - \hbox {BLR}_\mathrm{Param.~3}}{\hbox {BLR}_\mathrm{Param.~1}} \cdot 100, \end{aligned}$$

where in this case the comparison is between the BLR results from the parameterizations 1 and 3. Positive $\delta \hbox {BLR}$ values describe the relative amount of improvement of the BLR in percent if the ray-traced delays are applied to the VLBI analysis compared to not applying them.

In order to compare only reliable BLR results, baselines with a weighted and unweighted BLR value of more than 10 cm in both compared analysis solutions are removed from the comparison.

#### Comparing BLR results from analysis solutions with tropospheric gradient estimation (param. 1 vs. param. 3)

In this first comparison, the impact of the ray-traced delays on the BLR is determined in the case of tropospheric gradient estimation within the VLBI analysis. For this task, the analysis solutions from the parameterizations 1 and 3 are compared in terms of their BLR results.

**Fig. 1:**
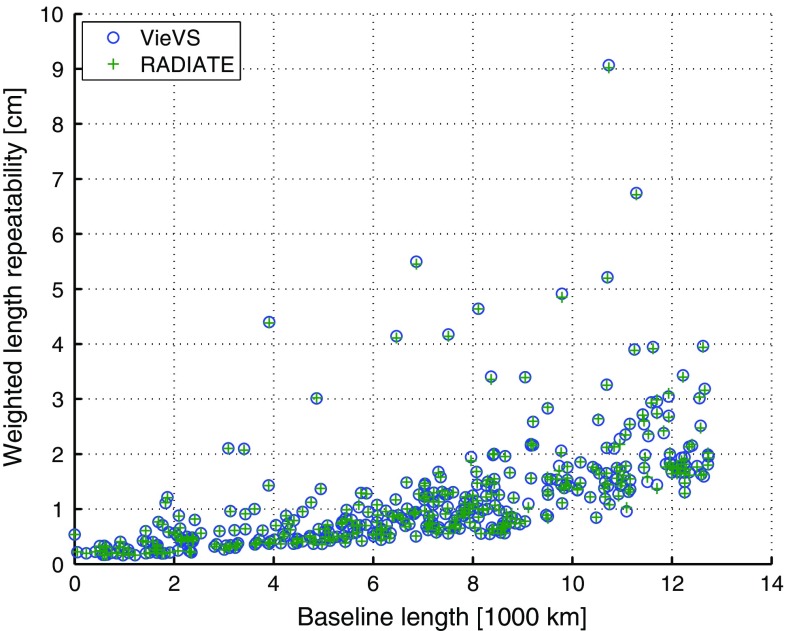
BLR of the 374 baselines derived from the VLBI analyses using the parameterizations 1 and 3. The blue circles denote the results from parameterization 1 (“VieVS”) without the application of ray-traced delays and the green pluses describe the results from parameterization 3 (“RADIATE”), which applies the ray-traced delays a priori to the analysis. Within the analyses of both solutions, tropospheric gradients have been estimated (Hofmeister 2016)

**Fig. 2:**
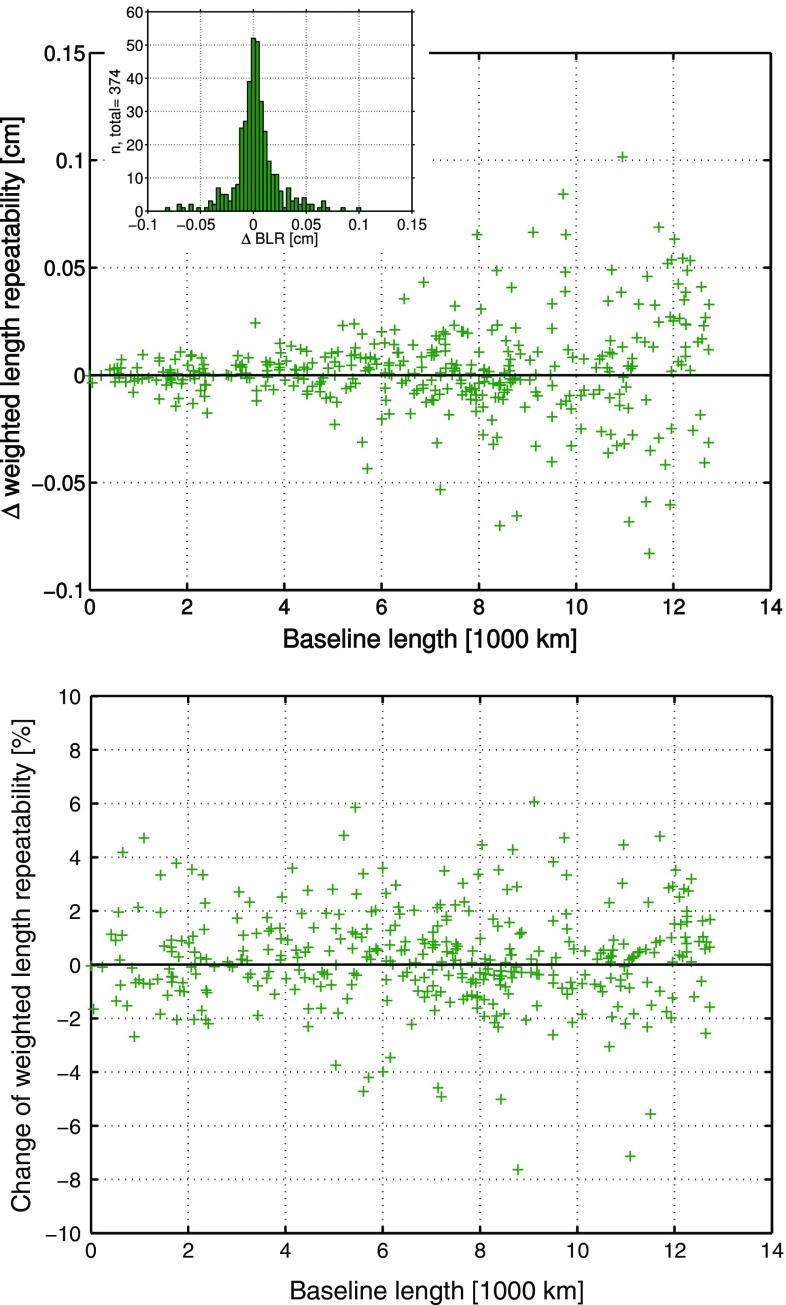
*Upper plot*: $\Delta \hbox {BLR}$ of the 374 baselines from parameterizations 1 (without applied ray-traced delays) and 3 (with applied ray-traced delays), where tropospheric gradients are estimated. Positive $\Delta \hbox {BLR}$ denote that the application of the ray-traced delays improves the BLR and thus the VLBI analysis. The histogram shows the distribution of the $\Delta \hbox {BLR}$. *Lower plot*: Relative change of the BLR ($\delta \hbox {BLR}$) as determined with Eq. (6). Positive $\delta \hbox {BLR}$ describe the percentage of relative improvement of the BLR by applying the ray-traced delays to the analysis (parameterization 3) compared to not applying them (parameterization 1) (Hofmeister 2016)

Figure 1 shows the absolute BLR results for the two parameterizations. The $\Delta \hbox {BLR}$ and $\delta \hbox {BLR}$ are presented in Fig. 2. The $\Delta \hbox {BLR}$ show that there is in general only little difference between the solutions due to the small values at the sub-mm level, but the relative BLR changes indicate that the application of the ray-traced delays positively impacts the improved baselines by a larger amount than it degrades the remaining baselines. In total, the BLR results of 374 different baselines of 44 stations are compared. In the mean, there is no impact on the BLR if the ray-traced delays are applied to an analysis, which includes the estimation of tropospheric gradients. Nevertheless, 55.9% of the baselines, i.e., 209 of 374, are improved at the sub-mm level in terms of their BLR if the ray-traced delays are used. There is also a slight average relative improvement of the BLR of 0.2% compared to not applying the ray-traced delays.

#### Comparing BLR results from analysis solutions without tropospheric gradient estimation (param. 2 vs. param. 4)

The second comparison takes a look at the impact of the ray-traced delays in the case of no tropospheric gradient estimation within the VLBI analysis and no instead a priori model usage. Thus, the analysis solutions from the parameterizations 2 and 4 are compared in terms of their BLR results.

**Fig. 3:**
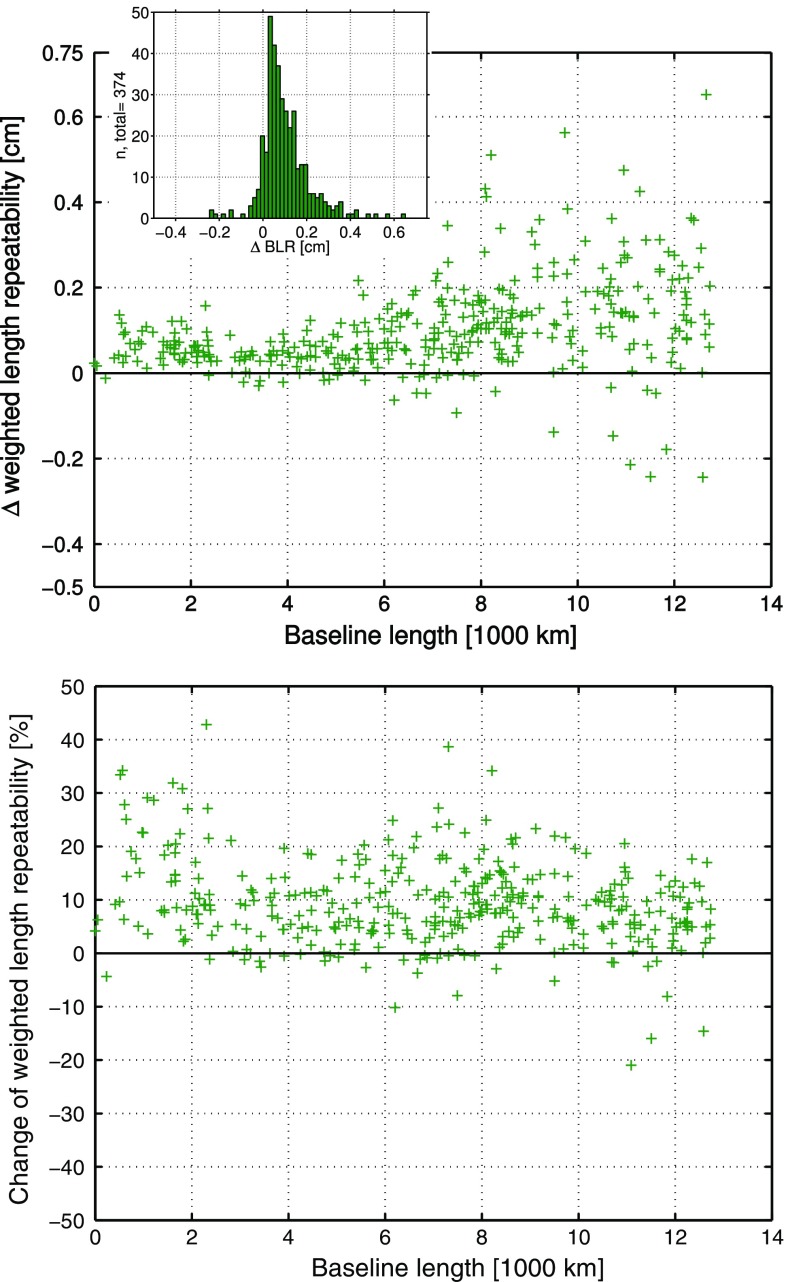
*Upper plot*: $\Delta \hbox {BLR}$ of the 374 baselines from parameterizations 2 (without applied ray-traced delays) and 4 (with applied ray-traced delays), where tropospheric gradients are not estimated. Positive $\Delta \hbox {BLR}$ denote that the application of the ray-traced delays improves the BLR and thus the VLBI analysis. The *y*-axis range is set five times as wide as in the corresponding plot of Fig. 2. The histogram shows the distribution of the $\Delta \hbox {BLR}$. *Lower plot*: Relative change of the BLR ($\delta \hbox {BLR}$). Positive $\delta \hbox {BLR}$ describe the percentage of relative improvement of the BLR by applying the ray-traced delays to the analysis (parameterization 4) compared to not applying them (parameterization 2). The *y*-axis range is set five times as wide as in the corresponding plot of Fig. 2 (Hofmeister 2016)

This comparison is based on the same 374 different baselines of 44 stations, which have already been investigated in the previous assessment in Sect. 3.3.1. The $\Delta \hbox {BLR}$ and $\delta \hbox {BLR}$ are displayed in Fig. 3.

Looking at the comparison results, it is obvious that the impact of the ray-traced delays on the VLBI analysis is increased if no tropospheric gradients are estimated within the VLBI analysis. The BLR of 90.6% of the baselines, i.e., 339 of 374, is improved due to the application of the ray-traced delays to the VLBI analysis. On average, the BLR is better by 1 mm and relatively improved on average by 9.3% compared to not applying the ray-traced delays. This significant enhancement of the BLR is due to the fact that the ray-traced delays implicitly provide the tropospheric gradient information. Since the gradients are not estimated for the compared solutions, their information is missing in the analysis if not introduced by the ray-traced delays. Thus, the application of the ray-traced delays is especially beneficial if no gradients are estimated.

#### Implications of no additional tropospheric gradient estimation if ray-traced delays are applied to the analysis in comparison with a standard VLBI analysis (param. 4 vs. param. 1)

It is investigated if it is sufficient to solely apply the ray-traced delays without additional tropospheric gradient estimation within the analysis since the ray-traced delays implicitly introduce the tropospheric gradient information. Therefore, the BLR results from an analysis with applied ray-traced delays, but without explicit tropospheric gradient estimation, i.e., the results from using parameterization 4, are compared to the BLR results of a standard VLBI analysis, i.e., the results from using parameterization 1, which is always used as a reference for the analysis accuracy in this work.

Based on this comparison, it is investigated if the applied ray-traced delays can solely and thus implicitly provide accurately the tropospheric gradient information needed within the analysis in order to deliver good BLR results, i.e., at least comparable to a standard VLBI analysis with tropospheric gradient estimation, but without the application of ray-traced delays.

Again the identical 374 different baselines of 44 stations are investigated in this comparison, which have already been assessed in the comparisons of the Sects. 3.3.1 and 3.3.2. In terms of the weighted BLR, which is used for the assessments in all presented comparisons, it shows that only 41.4% of the baselines have a better BLR if the ray-traced delays are applied and no tropospheric gradients are estimated within the analysis, i.e., using parameterization 4, compared to not using ray-traced delays but estimating tropospheric gradients, i.e., parameterization 1. The BLR of the solution from parameterization 4 is on average larger by 0.7 mm. Comparing the unweighted BLR more baselines benefit from parameterization 4 since 54% have a better BLR than in case of the standard VLBI analysis, i.e., using parameterization 1. Also in terms of the unweighted BLR there is on average a degradation if parameterization 4 is used, but this can be explained by the fact that the maximum BLR degradation is 12.7 cm, whereas the maximum BLR improvement is only 1.9 cm. Thus, the median, which shows an improvement of 1.3 mm if parameterization 4 is used, is more reliable in this case.

**Table 5:** Settings for determining the global solutions and TRF solutions as used together with the results of one certain single-session analysis from the different parameterizations 1–4

Global solution settings	
Station coordinates and velocities	Estimated at the epoch J2000.0
TRF determination settings	
Datum definition	12 Helmert parameters (No-Net-Translation (NNT) and No-Net-Rotation (NNR) conditions for positions and velocities)
Datum stations	11 well globally distributed stations: KOKEE, ALGOPARK, WESTFORD, FORTLEZA, NYALES20, ONSALA60, WETTZELL, MATERA, HARTRAO, SESHAN25 and HOBART26. Most of them cover almost the complete time interval of sessions in the global solution
Reduced stations	Stations participating in less than 10 sessions or with less than 2 years observation time span are reduced, i.e., these stations are not part of the global solution and estimated only session-wise. Furthermore, stations with unreliable a priori coordinates or with large formal coordinate errors in a test global solution are excluded
Station discontinuities	Treatment of station discontinuities, i.e., breaks in station positions. Station velocities are kept constant at specific stations with breaks caused by antenna repairs
Station velocity ties	Introduction of velocity ties for co-located sites

In conclusion, it can be stated that the BLR of some baselines is better if the ray-traced delays are applied to a VLBI analysis, which does not include a tropospheric gradient estimation, compared to a standard VLBI analysis solution. In such cases, the implicit tropospheric gradient information of the ray-traced delays is accurate enough to be used without additionally estimating it. Other baselines do need the additional tropospheric gradient estimation within the analysis to get the optimal BLR.

Earlier research on the application of ray-traced delays to the VLBI analysis of the Continuous VLBI Campaign 2011 (CONT11) showed that if tropospheric gradients but also ZWD are not estimated, the application of ray-traced delays is not enough to reach the level of BLR of a standard VLBI analysis, which estimates both the ZWD and the tropospheric gradients. All investigated baselines have been deteriorated in terms of their BLR. The mean BLR deterioration has been about 2 cm in this earlier research. This result leads to the conclusion that ZWD estimation is needed also in case of using ray-traced delays within the analysis.

### Influence of ray-traced delays on the terrestrial reference frame

Besides the BLR, the solution of a TRF is another important assessment domain for the impact of the ray-traced delays. For the determination of TRF solutions from the single-session analysis solutions from the parameterizations 1–4, it is necessary to estimate so-called global solutions. This task is again carried out with the software VieVS. The settings for the global solution estimation and the TRF determination are described in Table 5 and used for each single-session analysis solution from the different parameterizations.

The resulting TRF solutions are denoted according to the analysis parameterizations, which have been used for determining the underlying single-session solutions. Each TRF solution contains the same 46 stations.

The station coordinates of the individual TRF solutions have formal errors due to the estimation process within the global solution. In order to have a key parameter for assessing the overall accuracy of a station position, the so-called mean coordinate error $\sigma _\mathrm{XYZ}$ is determined using the individual component errors $\sigma _{X}$, $\sigma _{Y}$ and $\sigma _{Z}$ of the geocentric Cartesian coordinates *X*, *Y* and *Z* of the estimated station position. The mean coordinate error of a station is calculated as

7 $$\begin{aligned} \sigma _\mathrm{XYZ} = \sqrt{ \frac{\sigma _{X}^2 + \sigma _{Y}^2 + \sigma _{Z}^2}{3} }. \end{aligned}$$

The determined TRF solutions are again compared pairwise. In each comparison, one TRF is based on the analysis results from not applying ray-traced delays and one TRF is found from the analysis results from applying the ray-traced delays.

The assessments of the impact of the ray-traced delays on the estimated TRF solution are on one side based on the resulting station coordinates, i.e., on the displacement of a station compared to a TRF solution from an analysis without applied ray-traced delays, and on the other side based on the transformation parameters between the compared TRF.

In order to better ascertain the impact of the ray-traced delays, the station coordinate differences are transformed from the geocentric Cartesian coordinate system to local topocentric coordinate systems depending on the individual station position. From these transformations, the coordinate differences in the North ($\Delta N$), East ($\Delta E$) and up ($\Delta U$) components of each station are received.

Besides the separate component-wise representation of the coordinate differences also the combined displacement $\Delta P$ of a station in the horizontal plane is determined. This is done by the evaluation of

8 $$\begin{aligned} \Delta P = \sqrt{\Delta N^2 + \Delta E^2}. \end{aligned}$$

The second assessment of the impact of the ray-traced delays on the determined TRF is based on the transformation parameters between a TRF determined from no application of ray-traced delays to the VLBI analysis and a TRF estimated with the application of the ray-traced delays to the analysis.

The fundamental concept and equations for the TRF transformation used here in this work can be found in Petit and Luzum (2010). The 14 Helmert transformation parameters, which describe the station position transformation by 7 parameters and the station velocity transformation also through 7 parameters, are estimated in a least-squares adjustment. The fundamental transformation equation for the station position transformation is given by

9 $$\begin{aligned} \mathbf {X_{target}} = \mathbf {T} + (1+m) \cdot \mathbf {\underline{R}} \cdot \mathbf {X_{reference}}, \end{aligned}$$

where $\mathbf {X_{target}}$ denotes the station coordinate vector in the target TRF, $\mathbf {X_{reference}}$ is the station coordinate vector in the reference TRF, $\mathbf {T}$ depicts the translation vector, *m* is the scale and $\mathbf {\underline{R}}$ is the rotation matrix containing the rotation angles $\omega _i$ around the coordinate axes. From this basic equation also the station velocity transformation can be found through the temporal derivation of Eq. (9).

In order to be able to carry out a least-squares adjustment, the transformation equations for the station position and velocity need to be linearized. A detailed description of the steps from the position transformation equation across the velocity transformation equation toward the least-squares adjustment for determining the 14 Helmert transformation parameters is given in Hofmeister (2013, 2016).

The adjustment delivers the 7 position transformation parameters consisting of the translations $T_X$, $T_Y$ and $T_Z$, the rotation angles $\omega _X$, $\omega _Y$ and $\omega _Z$ and the scale *m* and the 7 temporal derivatives of the position transformation parameters for the description of the velocity transformation. These can be denoted accordingly as $\dot{T}_X$, $\dot{T}_Y$, $\dot{T}_Z$, $\dot{\omega }_X$, $\dot{\omega }_Y$, $\dot{\omega }_Z$ and $\dot{m}$.

For the least-squares adjustment, a certain set of tie points needs to be introduced, describing each station position and velocity in the two TRF that should be connected. Following Böckmann et al. (2010) it makes sense to check the results of the transformation parameters by evaluating them with different tie point sets in order to assess the robustness of the solutions. Therefore, three different tie point sets are used in this work:

1. $${\textit{Datum \,stations}}$$
  This tie point set contains only the 11 datum stations of the TRF. Note that each TRF solution in the presented research has the same datum stations.
2. $${\textit{Stations} \,\textit{with} \,\sigma _\mathrm{XYZ}<{4}~\hbox {mm}}$$
  The corresponding tie point set contains only those TRF stations, which have a mean coordinate error ${\sigma _\mathrm{XYZ}}$ of below 4 mm in a specific TRF solution as determined with Eq. (7). If the transformation parameters are determined between TRF 1 and TRF 3 then the tie point set is defined by all stations with ${\sigma _\mathrm{XYZ}<{4}~\hbox {mm}}$ in TRF 3, which are 44 stations. For the estimation of the transformation parameters between TRF 2 and TRF 4, the tie point set is defined by all stations with $\sigma _\mathrm{XYZ}<{4}~\hbox {mm}$ in TRF 4, which are again the same 44 stations.
3. $${\textit{All\, stations}}$$
  This tie point set contains all stations, which are part of the TRF. Since all TRF solutions contain the same 46 stations, all realizations of this tie point set used for the estimation of the transformation parameters are identical.

#### Comparing TRF solutions from analysis results which included tropospheric gradient estimation (param. 1 vs. param. 3)

In this first comparison, the impact of the ray-traced delays on the TRF if applied to a standard VLBI analysis is investigated. Thus, the tropospheric gradient estimation has been included in the analysis. From the single-session solutions of the parameterizations 1 and 3, TRF 1 and TRF 3 have been determined in global solutions and are compared to reveal the kind and size of the ray-traced delay impact.

**Fig. 4:**
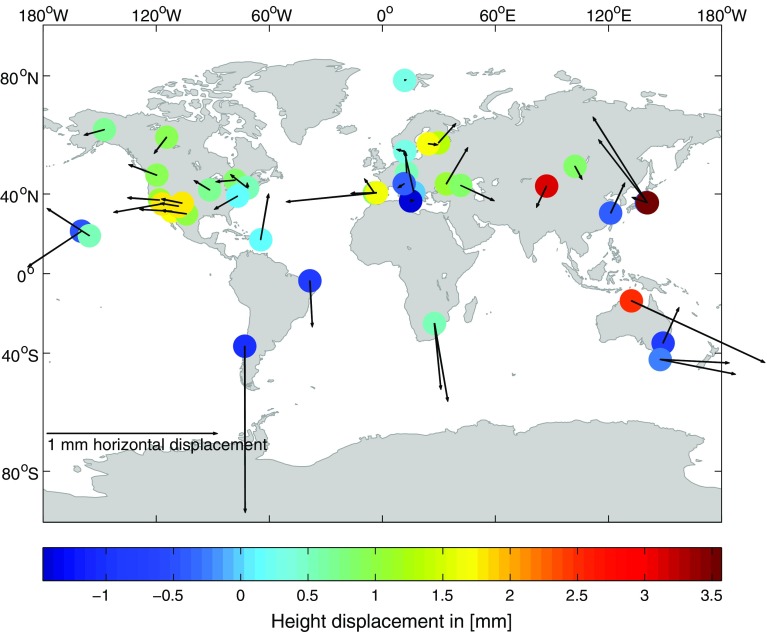
Horizontal and vertical displacements of the 44 stations with $\sigma _\mathrm{XYZ}<{4}~\hbox {mm}$ in TRF 3 due to the application of the ray-traced delays to the VLBI analysis. Differences in the station positions are computed as TRF 3–TRF 1 at the epoch J2000.0. Tropospheric gradients have been estimated within the analyses (Hofmeister 2016)

Figure 4 depicts the impact of the ray-traced delays on the TRF if tropospheric gradients are estimated within the VLBI analysis and shows the effect on the horizontal and vertical components of the 44 stations with $\sigma _\mathrm{XYZ}<{4}~\hbox {mm}$ in TRF 3.

**Fig. 5:**
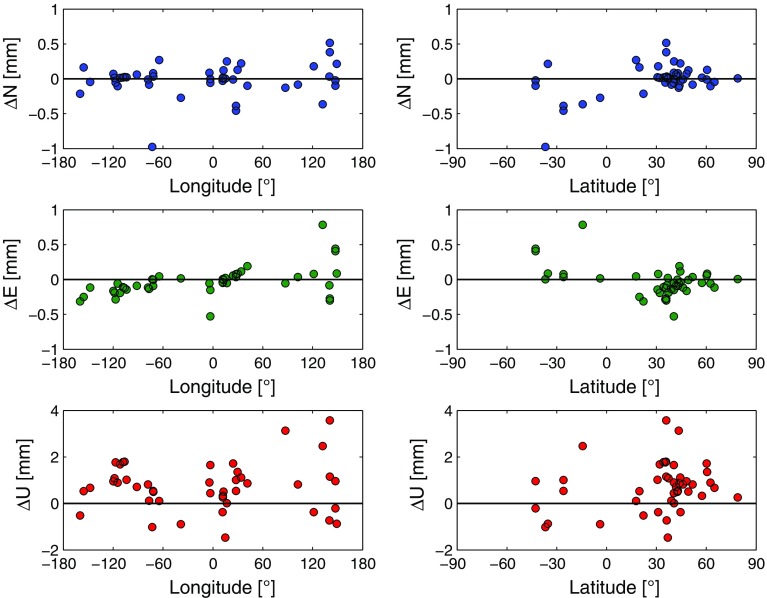
Component-wise representation of the local coordinate differences of the 44 stations with $\sigma _\mathrm{XYZ}<{4}~\hbox {mm}$ in TRF 3 due to the application of the ray-traced delays to the VLBI analysis. The coordinate differences are computed as TRF 3–TRF 1 at the epoch J2000.0. Tropospheric gradients have been estimated within the analyses. *Plots on the left side*: $\Delta N$, $\Delta E$ and $\Delta U$ presented with respect to the stations’ longitudes. *Plots on the right side*: $\Delta N$, $\Delta E$ and $\Delta U$ displayed with respect to the stations’ latitudes (Hofmeister 2016)

The use of this set of stations ensures that only reliable coordinate estimates, i.e., stations, are taken into account. Therefore the results of the current TRF comparison provided in the following are always determined with respect to this set of stations unless stated differently. In the horizontal domain, the stations seem to be shifted outwards from Central Europe due to the application of the ray-traced delays. This trend is nevertheless not significant due to the small sizes of the displacements. For a more detailed insight into the effect of the ray-traced delays on the station positions, Fig. 5 shows the local coordinate differences $\Delta N$, $\Delta E$ and $\Delta U$ with respect to station latitudes and longitudes.

Also from the component-wise representation no significant latitude or longitude dependence of the impact of the ray-traced delays can be derived. The averages of the coordinate differences are: $\overline{\Delta N} = -0.0$ mm, $\overline{\Delta E} = -0.0$ mm and $\overline{\Delta U} = 0.7$ mm. The average horizontal displacement is $\overline{\Delta P} = 0.2$ mm.

The maximum horizontal displacement with respect to all stations contained in the TRF is 1.1 mm at the station YARRA12M, i.e., there is no significant effect of the ray-traced delays on the horizontal station positions if tropospheric gradients are estimated within the VLBI analysis. The station height displacements are mostly between ±2 mm and overall the height components show the tendency of an uplift if ray-traced delays are applied to the analysis.

**Fig. 6:**
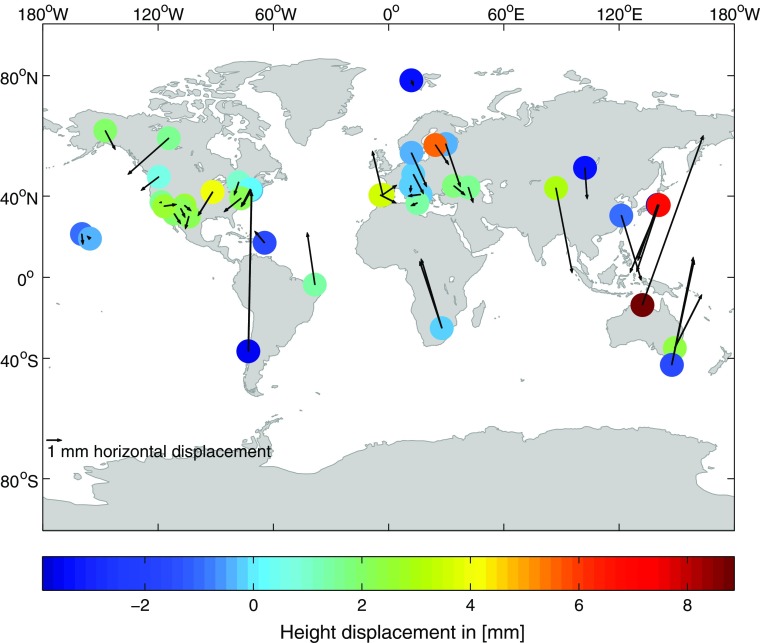
Horizontal and vertical displacements of the 44 stations with $\sigma _\mathrm{XYZ}<{4}~\hbox {mm}$ in TRF 4 due to the application of the ray-traced delays to the VLBI analysis. Differences in the station positions are computed as TRF 4–TRF 2 at the epoch J2000.0. Tropospheric gradients have not been estimated within the analyses (Hofmeister 2016)

The transformation parameters between TRF 1 and TRF 3 reveal that the application of the ray-traced delays does not affect the TRF in general. Independent of the used tie point set in the least-squares adjustment sub-mm translations and as rotations are determined for the position transformation. The scale of the TRF is changed by only 0.1 ppb in case of the tie point set of stations with $\sigma _\mathrm{XYZ}<{4}~\hbox {mm}$ in TRF 3. If only the datum stations are used as tie points, the change is even 0.0 ppb. The temporal derivatives of the position transformation parameters are too small to be relevant. The transformation parameter estimates from the different tie point sets deliver similar values, thus providing the desired robustness check.

In general, the compared TRF solutions can be seen as almost equal. Only with regard to the station heights, there is a slight influence due to the application of the ray-traced delays.

#### Comparing TRF solutions from analysis results which did not include tropospheric gradient estimation (param. 2 vs. param. 4)

In the second comparison, the impact of the ray-traced delays on the TRF if applied to the VLBI analysis is investigated in the case of no tropospheric gradient estimation within the analysis. Again two TRF solutions have been determined from the results of different single-session analyses. TRF 2 has been determined using the results from parameterization 2, i.e., without usage of ray-traced delays, and TRF 4 has been created from the solutions of parameterization 4, i.e., with application of the ray-traced delays.

Figure 6 shows the impact of the ray-traced delay application on the TRF if no tropospheric gradients are estimated within the VLBI analysis. The horizontal and vertical displacements of the 44 stations with $\sigma _\mathrm{XYZ}<{4}~\hbox {mm}$ in TRF 4 are depicted.

**Fig. 7:**
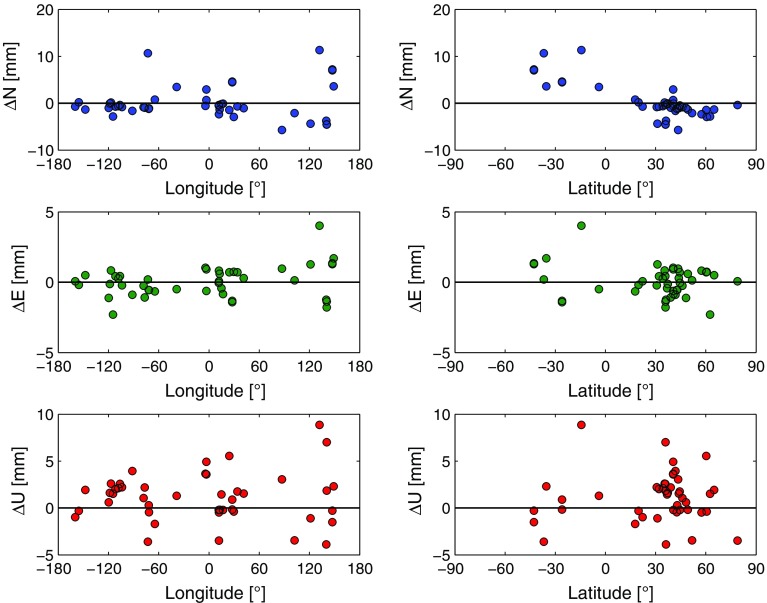
Component-wise representation of the local coordinate differences of the 44 stations with $\sigma _\mathrm{XYZ}<{4}~\hbox {mm}$ in TRF 4 due to the application of the ray-traced delays to the VLBI analysis. The coordinate differences are computed as TRF 4–TRF 2 at the epoch J2000.0. Tropospheric gradients have not been estimated within the analyses. *Plots on the left side*: $\Delta N$, $\Delta E$ and $\Delta U$ presented with respect to the stations’ longitudes. *Plots on the right side*: $\Delta N$, $\Delta E$ and $\Delta U$ displayed with respect to the stations’ latitudes (Hofmeister 2016)

This set of stations is exactly the same as in the previous comparison in Sect. 3.4.1. The results of the current TRF comparison provided in the following are always determined with respect to this set of stations unless stated differently. Due to the application of the ray-traced delays, the horizontal station positions are shifted and there is a clear pattern visible. Stations in the northern hemisphere are displaced toward the South, and stations in the southern hemisphere are displaced toward the North. This effect of the ray-traced delays can be explained by the fact that they implicitly introduce the missing tropospheric gradient information to the analysis. In principle, both analysis solutions used for the determination of the two compared TRF are lacking the tropospheric gradient information since the gradients are not explicitly applied a priori or estimated. Through the application of the ray-traced delays to one of the solutions, i.e., to the one from parameterization 4, the tropospheric gradient information is though introduced. Thus, the horizontal station displacements, visible in Fig. 6, are reasonable. A detailed component-wise investigation of the influence of the ray-traced delays on the estimated stations of the TRF is presented in Fig. 7, which shows the local coordinate differences $\Delta N$, $\Delta E$ and $\Delta U$ with respect to station latitudes and longitudes.

The component-wise representation confirms the above explained influence of the ray-traced delays on the horizontal station positions. The uppermost plot on the right side of Fig. 7, i.e., the plot of the $\Delta N$ with respect to station latitudes, shows that all stations in the southern hemisphere have a positive $\Delta N$ and almost all stations in the northern hemisphere have a negative $\Delta N$. The other components do not show significant trends with respect to the latitudes or longitudes. The averages of the coordinate differences are: $\overline{\Delta N} = 0.1$ mm, $\overline{\Delta E} = 0.0$ mm and $\overline{\Delta U} = 1.1$ mm. The average horizontal displacement is $\overline{\Delta P} = 2.7$ mm.

The impact of the ray-traced delays on the TRF if no tropospheric gradients are estimated within the analysis is significantly increased with respect to the horizontal station positions compared to the previous comparison described in Sect. 3.4.1. Also the impact on the station heights is increased. Most of the station heights are changed by ±4 mm.

Considering the transformation parameters between TRF 2 and TRF 4, it can be stated that the three different tie point sets used within the least-squares adjustments for the parameter determination deliver similar results, which provides confidence that the estimates are robust. The compared TRF solutions are very similar regarding the transformation parameters. For the position transformation the translations are still at the sub-mm level, but slightly increased compared to the values of the comparison in Sect. 3.4.1. The same holds true for the rotation angles, where now the angles $\omega _X$ and $\omega _Y$ are increased to values of around 30 as to 40 as from the level of a few as. The scale of the TRF is again only affected by 0.1 ppb if the tie point set of stations with $\sigma _\mathrm{XYZ}<{4}~\hbox {mm}$ in TRF 4 is considered. If only the datum stations are taken into account for the transformation parameter estimation, the scale is oppositely influenced by -0.1 ppb. This follows from the fact that the average station height change of the datum stations is -0.4 mm, but due to the small sample size of stations not too much attention should be drawn to this particular result. Although the TRF is affected more than in Sect. 3.4.1 by the application of the ray-traced delays if no tropospheric gradients are estimated within the analysis, the scale change remains with 0.1 ppb the same. This can be explained by the increased translations and rotation angles, which compensate also for the increased station height changes besides the horizontal station position changes. Usually it would be expected that the station height changes can be directly transformed into scale changes. The temporal derivatives of the position transformation parameters are again too small to be significant.

From the assessed transformation parameters it is obvious that the compared TRF solutions are in general very similar, but looking at the station positions, there is a clear change of the TRF due to the application of the ray-traced delays, which implicitly introduce the missing tropospheric gradient information. Accordingly, this effect is especially visible in terms of the horizontal station positions, which are mostly affected by the tropospheric gradients.

## Comparison of the impact of ray-traced delays from RADIATE and from NASA GSFC on the VLBI analysis 

In this section, the performance of the ray-traced delays from program RADIATE is investigated in terms of a comparison to the performance of the ray-traced delays from the National Aeronautics and Space Administration Goddard Space Flight Center (NASA GSFC). The decision to compare to the NASA GSFC delays was made, because these have been the only other ray-traced delays derived from NWM data and available for the desired long time span. For the assessment, the ray-traced delays are applied to the VLBI analysis and the BLR are determined. In each analysis, which is again carried out using the VLBI software VieVS, either the ray-traced delays from RADIATE or those from NASA GSFC are applied. Based on the differences in the resulting BLR from using RADIATE or NASA GSFC ray-traced delays, conclusions to the performance differences can be drawn.

### Data for the research

For the assessment of the performance differences between the RADIATE and the NASA GSFC ray-traced delays, it is again necessary to define a suitable set of VLBI observational data. The utilized ray-traced delays as well as the chosen VLBI observational data set are described in the following.

#### Ray-traced tropospheric slant delays

The ray-traced delays from RADIATE, which are used for this assessment, are the same as described in Sect. 3.1.2.

The NASA GSFC ray-traced delays (see Eriksson and MacMillan 2016) have been taken from their service on the web page http://lacerta.gsfc.nasa.gov/tropodelays. Details on the determination of the NASA GSFC ray-traced delays, e.g., the used NWM data and the ray-tracing approach, can be found in Eriksson et al. (2014).

#### VLBI observational data

For the comparison of the analysis impact of the RADIATE and NASA GSFC ray-traced delays suitable VLBI observational data needed to be chosen. In principle, the initial data set defined in Sect. 3.1.1, which contains 2461 sessions^4^ in the time span 1999.0–2015.5, had been selected to be used also for the comparisons of this assessment since it provides a long time series. Unfortunately, the desired time span of the observational data is not fully covered by the ray-traced delays obtained from NASA GSFC. Since only ray-traced delays for sessions in the interval 2000.0–2015.1 were available, the observational data set had to be limited to this slightly shorter interval. Furthermore, for some sessions in this interval, which have been chosen to be part of the comparison data set, ray-traced delay data from NASA GSFC were missing. Thus, the observational data set has been limited to the interval 2000.0–2015.1, i.e., a time span of approximately 15 years, and reduced from 2461 to 2196 sessions, which are analyzed.

In order to determine the BLR only from reliable analysis results, again an exclusion of sessions with unreliable solutions is done prior to the BLR calculations in the same manner as in Sect. 3.1.1. In detail, this means that those sessions, which have in at least one of the carried out analyses in this work either a solution of the least-squares adjustment that is close to singular, an a posteriori standard deviation of unit weight of larger than 3 or at least one baseline in the solution with a formal error of its length estimate of more than 10 cm, are removed from the data set used for the BLR determination. A further exclusion of some sessions is necessary since the ray-traced delay data obtained from NASA GSFC do not always provide a ray-traced delay for every analyzed observation. If any of the needed ray-traced delays has been missing in the NASA GSFC data during the analysis, the respective session is excluded from the BLR determination in order to ensure that the BLR are estimated only from analysis results from pure ray-traced delay usage. The application of all of these described criteria leads to a reduction of the sessions, which are introduced to the BLR determination, from 2196 to 2085.

### VLBI analysis parameterization

For the analysis of the VLBI sessions, the software VieVS is used again. Two different analysis parameterizations are used to compare the performances, i.e., the differences in the impact of the ray-traced delays from RADIATE and from NASA GSFC. These parameterizations are the ones defined as parameterization 3 and parameterization 4 in Sect. 3.2, which apply the ray-traced delays to the analysis. Please note that despite the names of the two parameterizations, i.e., “RADIATE” and “RADIATE no gradients”, their settings are also meant to be used if the NASA GSFC ray-traced delays are applied to the analysis as done within this section. The Tables 2, 3 and 4 provide the details on the parameterizations.

The VLBI sessions are analyzed four times. At first two times using parameterization 3. Once with applying only RADIATE ray-traced delays and once with applying only NASA GSFC ray-traced delays. The same procedure is repeated with parameterization 4.

### Performance differences in baseline length repeatability

The BLR are determined separately for each of the four different single-session analysis solutions. The calculations are done again according to the method described in Sect. 3.3, i.e., also with the same approach for a correct trend reduction.

For the investigation of the performance differences between the ray-traced delays from RADIATE and from NASA GSFC, the differences in the BLR ($\Delta \hbox {BLR}$) are determined between the solutions from the same analysis parameterization, but derived from the application of the different ray-traced delays, i.e., one solution originates from the application of the NASA GSFC ray-traced delays and one solution originates from the application of the RADIATE ray-traced delays. According to the formalism of the computed differences, positive $\Delta \hbox {BLR}$ denote that the BLR is improved or better if the ray-traced delays from program RADIATE are applied to the analysis.

Again also the relative amount of change of the BLR ($\delta \hbox {BLR}$) is assessed. The calculations of the $\delta \hbox {BLR}$ are done according to the fundamental formalism presented by Eq. (6). Instead of applying the BLR results from two different analysis parameterizations to the equation, the BLR determined from using the same parameterization but from utilization of the two different ray-traced delay sources are applied. Positive $\delta \hbox {BLR}$ values describe the relative amount of improvement of the BLR in percent if the ray-traced delays from program RADIATE are applied to the VLBI analysis instead of applying the ray-traced delays from NASA GSFC.

To ensure that only reliable BLR results are compared, equal to Sect. 3.3, baselines with a weighted and unweighted BLR value of more than 10 cm in both compared analysis solutions are removed from the comparison.

#### BLR performance differences from analysis solutions with tropospheric gradient estimation (param. 3)

**Fig. 8:**
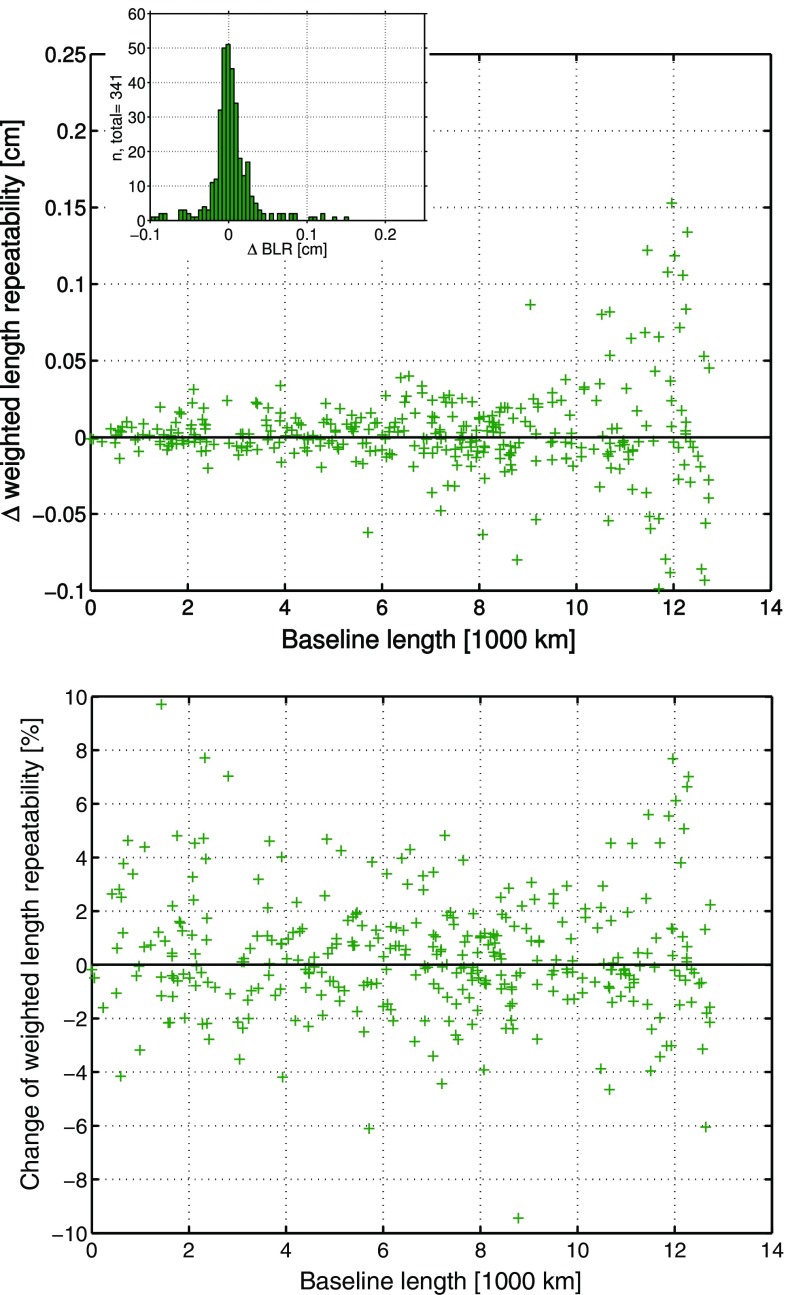
*Upper plot*: $\Delta \hbox {BLR}$ of the 341 baselines derived from the BLR of the VLBI analysis solutions from parameterization 3, where tropospheric gradients are estimated. Once the ray-traced delays from NASA GSFC and once the ray-traced delays from RADIATE have been applied a priori to the analysis. Positive $\Delta \hbox {BLR}$ denote that the application of the ray-traced delays from RADIATE improves the BLR. The histogram shows the distribution of the $\Delta {\hbox {BLR}}$. *Lower plot*: Relative change of the BLR ($\delta \hbox {BLR}$). Positive $\delta \hbox {BLR}$ describe the percentage of relative improvement of the BLR by applying the ray-traced delays from RADIATE to the analysis instead of applying the ray-traced delays from NASA GSFC (Hofmeister 2016)

At first, the performance differences between the ray-traced delays from RADIATE and NASA GSFC are determined based on the analysis with parameterization 3, i.e., if tropospheric gradients are estimated within the analysis. The $\Delta \hbox {BLR}$ and $\delta \hbox {BLR}$ are presented in Fig. 8.

The $\Delta \hbox {BLR}$ show that the compared solutions deliver very similar BLR results for the 341 different baselines of 41 stations. The majority of baselines shows differences of only ±0.5 mm. On average there is no difference in the BLR if ray-traced delays from RADIATE or from NASA GSFC are applied to analyses with included tropospheric gradient estimation. Nevertheless, it is the case that with 51.3% of the baselines, i.e., 175 of 341, slightly more than half of the baselines are improved in terms of their BLR if the RADIATE ray-traced delays are used. The relative changes in the BLR ($\delta \hbox {BLR}$) confirm the slightly better performance of the RADIATE ray-traced delays since there is on average a relative improvement of the BLR of 0.3% if the RADIATE ray-traced delays are applied to the analysis instead of the NASA GSFC ray-traced delays. Furthermore, it seems that those baselines, which are improved by the RADIATE ray-traced delays, have more benefit or improvement than there is degradation at the remaining baselines due to their usage.

*Side note* In the following, a short investigation is carried out on the agreement of the analysis results from applied ray-traced delays presented in this work to results published by Eriksson et al. (2014).

Eriksson et al. (2014) found an improvement of the BLR for 72%^5^ or 71%^6^ of the investigated baselines in terms the NASA GSFC ray-traced delays are applied to the analysis compared to the a priori use of tropospheric delays derived with the VMF1.

The comparison shown above in Sect. 4.3.1 revealed that the application of the ray-traced delays from RADIATE or those from NASA GSFC leads to similar results in terms of the BLR with a slightly better performance of the ray-traced delays from RADIATE. In Sect. 3.3.1, the impact of the application of the ray-traced delays from RADIATE to the VLBI analysis has been assessed in terms of a comparison against a standard VLBI analysis, which uses a priori tropospheric delays derived with the VMF1. This assessment included the tropospheric gradient estimation within the analysis every 2 h.^7^ As a result, it has been shown that 55.9% of the baselines are improved in terms of the BLR if the RADIATE ray-traced delays are applied to the analysis instead of using a priori tropospheric delays derived with the VMF1.

Thus, at a first glance this assessment result seems contradictory to the above-described findings of Eriksson et al. (2014) and the result of the comparison between the ray-traced delays from RADIATE and from NASA GSFC in this work as an improvement of the BLR at more than 70% of the baselines is expected based on these results. This contradiction can be resolved by a closer look at the analysis parameterizations used by Eriksson et al. (2014) for the derivation of their solutions. They estimated the tropospheric gradients within the analysis only every 6 h, whereas the parameterizations in this work here use an estimation interval of 2 h. This difference in the analysis parameterization clarifies the contradiction since the impact of the ray-traced delays on the analysis is significantly increased if there are fewer tropospheric gradient estimates available since the implicit gradient information of the ray-traced delays becomes then more important. This means that through the tropospheric gradient estimation interval used by Eriksson et al. (2014) the impact of the ray-traced delays is increased compared to the settings used in the research presented here. Additionally other analysis settings used by Eriksson et al. (2014) are different from those used in this work here. Also the observational data sets of Eriksson et al. (2014) are significantly different from the observation data utilized in the research presented here, which definitely impacts the percentage of baselines improved by the application of ray-traced delays.

#### BLR performance differences from analysis solutions without tropospheric gradient estimation (param. 4)

For the second assessment of the performance differences between the ray-traced delays from RADIATE and those from NASA GSFC parameterization 4 is chosen for the VLBI analysis, which means that no tropospheric gradients are applied a priori and different to the assessment in Sect. 4.3.1 also no tropospheric gradients are estimated within the analysis.

**Fig. 9:**
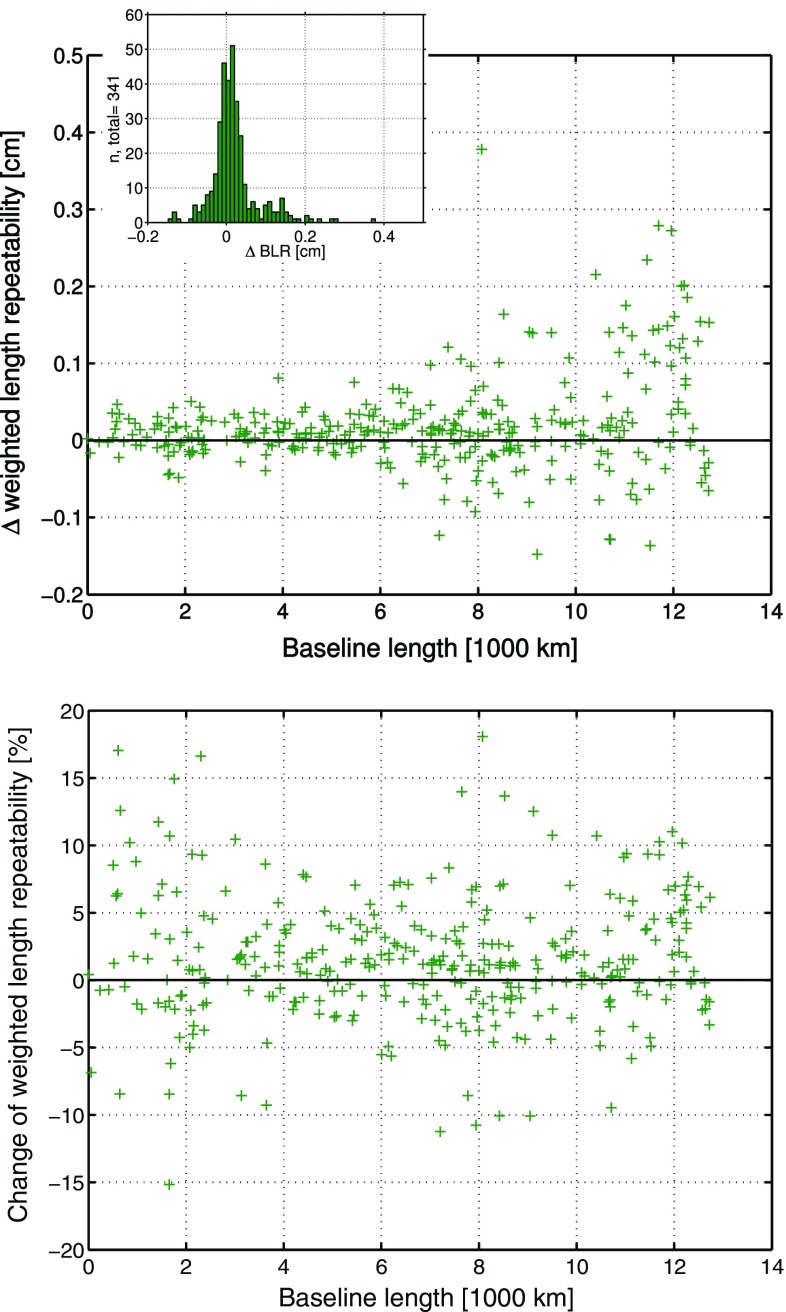
*Upper plot*: $\Delta \hbox {BLR}$ of the 341 baselines derived from the BLR of the VLBI analysis solutions from parameterization 4, where tropospheric gradients are not estimated. Once the ray-traced delays from NASA GSFC and once the ray-traced delays from RADIATE have been applied a priori to the analysis. Positive $\Delta \hbox {BLR}$ denote that the application of the ray-traced delays from RADIATE improves the BLR. The *y*-axis range is set twice as wide as in the corresponding plot of Fig. 8. The histogram shows the distribution of the $\Delta \hbox {BLR}$. *Lower plot*: Relative change of the BLR ($\delta \hbox {BLR}$). Positive $\delta \hbox {BLR}$ describe the percentage of relative improvement of the BLR by applying the ray-traced delays from RADIATE to the analysis instead of applying the ray-traced delays from NASA GSFC. The *y*-axis range is set twice as wide as in the corresponding plot of Fig. 8 (Hofmeister 2016)

The following comparison is based on the same 341 different baselines of 41 stations, which have already been investigated in the previous comparison in Sect. 4.3.1. The $\Delta \hbox {BLR}$ and $\delta \hbox {BLR}$ are displayed in Fig. 9.

From the $\Delta \hbox {BLR}$ results between the analysis solutions from parameterization 4 it is obvious that the performance differences between the ray-traced delays from RADIATE and those from NASA GSFC are increased compared to the previous comparison in Sect. 4.3.1. Most of the baselines have a $\Delta \hbox {BLR}$ between ±1 mm. Furthermore, significantly more baselines have a better BLR if the ray-traced delays from RADIATE are applied to the VLBI analysis. In detail, 63% of the baselines, i.e., 215 of 341, are then improved compared to the usage of the NASA GSFC ray-traced delays. On average the BLR is improved by 0.2 mm if the RADIATE ray-traced delays are applied to the VLBI analysis instead of the NASA GSFC ray-traced delays. A mean relative improvement of the BLR of 1.5% is reached by applying the RADIATE ray-traced delays instead of the NASA GSFC ray-traced delays. Also in the current comparison it is the case that the improvement of those baselines, which benefit from the application of the RADIATE ray-traced delays, is larger than the degradation of the remaining baselines due to them.

Since no a priori tropospheric gradients have been applied to the analysis and no estimation of the gradients has been done in case of the utilized parameterization 4, the analysis is depending on the tropospheric gradient information, which is implicitly introduced by the application of the ray-traced delays. Thus, it is necessary that the applied ray-traced delays accurately pass this information to the analysis. The better the tropospheric gradient information is introduced, the better the results of the BLR will be. Looking at the above-described performance differences between the ray-traced delays from RADIATE and those from NASA GSFC, it seems that the RADIATE ray-traced delays implicitly contain the more accurate tropospheric gradient information compared to the NASA GSFC ray-traced delays. The impact of the tropospheric gradients is larger at shorter baselines. The $\Delta \hbox {BLR}$ shown in the upper plot of Fig. 9 reveal that especially at such shorter baselines the RADIATE ray-traced delays perform better than the NASA GSFC ray-traced delays.

The observed performance differences between the ray-traced delays from RADIATE and those from NASA GSFC may mainly evolve from the different NWM, which are used for the ray-traced delay determination. According to Eriksson et al. (2014) the NASA GSFC ray-traced delays are determined by utilizing the NASA GSFC GMAO GEOS-5 FP-IT^8^ NWM, which is a terrain following model. As noted in Sect. 3.1.2 the RADIATE ray-traced delays are determined by using ECMWF pressure level data as NWM data input.

## Conclusions

In the presented research, the impact of the application of ray-traced delays to the VLBI analysis has been investigated. For the general investigations of the impact, the ray-traced delays from program RADIATE (see Hofmeister 2016) have been used. Additionally their performance has been assessed in comparison with ray-traced delays from NASA GSFC.

It is revealed that the utilization of the ray-traced delays in an analysis with included tropospheric gradient estimation delivers similar BLR results compared to a standard VLBI analysis, which also includes the tropospheric gradient estimation but which does not use ray-traced delays. On average the assessed BLR results are equal, but the application of the ray-traced delays nevertheless improves 55.9% of the baselines, i.e., more than the half, in terms of the BLR at the level of sub-mm. With respect to the TRF, there is no significant impact of the application of the ray-traced delays if tropospheric gradients are estimated within the analysis. The ray-trace TRF solution is very close to the TRF of a standard solution without the utilization of ray-traced delays if the determined transformation parameters between the frames are considered. No significant impacts on the scale of the frame (only 0.1 ppb) or on the horizontal station positions are found. Only with regard to the station heights, a slight average uplift tendency of 0.7 mm due to the ray-traced delays is derivable with respect to the reliable station set with $\sigma _\mathrm{XYZ}<{4}~\hbox {mm}$ in TRF 3.

The impact of the ray-traced delays on the VLBI analysis is significantly more evident if there is no estimation of tropospheric gradients within the analysis. In such a case, the analysis solution is considerably improved by the application of the ray-traced delays. The BLR is improved on average by 1 mm and a better BLR is reached at 90.6% of the baselines. A mean relative improvement of the BLR of 9.3% is reached compared to not applying the ray-traced delays. These results lead to the conclusion that the tropospheric gradient information, which is implicitly contained in the ray-traced delays, is extremely important and beneficial if the gradients are not estimated within the analysis. Considering the TRF solutions derived without tropospheric gradient estimation during the analysis, it can be stated that the frames are very similar in terms of the transformation parameters between the TRF solution from the analysis without the application of ray-traced delays and the TRF solution from the analysis with applied ray-traced delays. Thus, the impact of the ray-traced delays on the TRF is quite small with this respect. Nevertheless, the impact of the ray-traced delays is evident in terms of the station positions. The implicitly introduced tropospheric gradient information from the ray-traced delays leads to a shift of the horizontal station positions by 2.7 mm on average with respect to the station set with $\sigma _\mathrm{XYZ}<{4}~\hbox {mm}$ in TRF 4. On average the stations in this set are uplifted by 1.1 mm. The scale of the frame is not significantly influenced by the application of the ray-traced delays to the VLBI analysis. It is changed by only 0.1 ppb if the tie point set of stations with $\sigma _\mathrm{XYZ}<{4}~\hbox {mm}$ in TRF 4 is used for the transformation parameter determination.

A further investigation revealed that it is dependent on the baseline if the application of the ray-traced delays without additional tropospheric gradient estimation within the analysis delivers a better BLR result than received from a standard VLBI analysis with included tropospheric gradient estimation but no application of ray-traced delays. This means that dependent on the baseline the tropospheric gradient estimation within the analysis may be needed in addition to the applied ray-traced delays to get the optimal BLR.

The comparison of the VLBI analysis performance from applying the ray-traced delays from program RADIATE to the performance from utilizing the ray-traced delays from NASA GSFC revealed that if tropospheric gradients are estimated within the analysis both the RADIATE and the NASA GSFC ray-traced delays deliver on average equal BLR results. Nevertheless, slightly more than half of the baselines, i.e., 51.3%, have a better BLR at sub-mm difference if the RADIATE ray-traced delays are used and the BLR is on average relatively improved by 0.3% compared to the NASA GSFC ray-traced delay application.

If there is no tropospheric gradient estimation within the VLBI analysis the RADIATE ray-traced delay application delivers for 63% of the baselines a better BLR result. On average the BLR is better by 0.2 mm and the average relative improvement compared to the application of the NASA GSFC ray-traced delays is 1.5%. Since the ray-traced delays implicitly introduce the tropospheric gradient information it can be concluded from this comparison result that the RADIATE ray-traced delays supply the more accurate gradient information. Especially at the shorter baselines, where the tropospheric gradients have an increased impact on the BLR, the RADIATE ray-traced delays perform better than the NASA GSFC ray-traced delays.

The reason for the observed performance differences between the RADIATE and the NASA GSFC ray-traced delays and thus between the corresponding BLR results may mainly evolve from the different NWM, which are used for the determination of the corresponding ray-traced delays, and not from the utilized ray-tracing approach.

The comparison of the performance of the RADIATE ray-traced delays with the performance of the NASA GSFC ray-traced delays serves as a highly successful validation and assessment of the RADIATE ray-traced delays and provides the indication of their good accuracy.

From the results presented in this research, it can be concluded that the application of ray-traced delays to the VLBI analysis is a useful alternative method for the a priori correction of the tropospheric effects acting on the VLBI observations compared to the common way, which utilizes mapping functions and zenith delays to determine the a priori tropospheric slant delays. The tropospheric gradient information implicitly contained in the ray-traced delays is valuable for the VLBI analysis and thus ray-traced delays can significantly improve analysis solutions without tropospheric gradient estimation, but also if tropospheric gradients are estimated, the analysis solution slightly benefits from the application of ray-traced delays. The amount of this improvement is mainly dependent on the estimation interval of the tropospheric gradients. We expect that longer intervals increase the positive impact of the ray-traced delays.

## Outlook

Since program RADIATE can be used to determine ray-traced delays not only for real VLBI observations, but also for simulated observations, it is possible to probe the atmosphere in terms of ray-tracing and use the gathered information as main basis to establish new tropospheric delay models. Thus, new or improved mapping functions and tropospheric gradient models can be created. This development is and has already been carried out at the Research Group Advanced Geodesy of the Department of Geodesy and Geoinformation at Technische Universität Wien by applying ray-traced delays from program RADIATE. Further developments in this area of scientific research are currently ongoing at the research group.

Program RADIATE uses meteorological data from post-processing NWM in order to receive the most accurate data, because they agree with observations. Since it is a future goal in VLBI to head toward near real-time analysis as it is already done for selected intensive sessions, meteorological data from forecast NWM can be applied in order to be able to provide the ray-traced delays in advance and thus to enable their application to the near real-time VLBI analysis.

The application of ray-traced delays is not limited to VLBI observations and could be used for correction of observations from other space geodetic techniques. GNSS observations are an important area of application, but concerning the large amount of observational data, a complete calculation of ray-traced delays for all observations will require a much more substantial computational effort than for VLBI.

The determination of ray-traced delays is also possible for the observations of satellite laser ranging (SLR) and lunar laser ranging (LLR). Concerning the application of program RADIATE for this task, adaptations of the ray path and delay calculations are required due to the different signal frequencies of these space geodetic techniques compared to VLBI signals. The determination of ray-traced delays for SLR observations has already been tested successfully with a correspondingly reconfigured version of program RADIATE.

The RADIATE ray-traced delays and further products from the ray-tracing will be made available to interested users and institutions.

## Footnotes

1
$= \sqrt{\chi ^2 / n_\mathrm{f}}$. $\chi ^2$ is the Chi-squared and $n_\mathrm{f}$ is the degree of freedom of the solution.

2 Due to the reduced number of total pressure levels of the ECMWF operational NWM at epochs before 01.01.2008, this NWM is substituted by the ERA-Interim NWM at earlier epochs.

3 An investigation on the optimal horizontal resolution of the NWM to be used for the operational ray-tracing processing can be found in Hofmeister (2016). A condensed form of the research on the impact of the horizontal resolution of the NWM on the ray-traced delays and the subsequent VLBI analysis is given in Hofmeister et al. (2015). In this investigation, it is shown that the use of the NWM data with a horizontal resolution of $1{^\circ } \times 1{^\circ }$ for the determination of the ray-traced delays, which are then applied to the VLBI analysis, leads to comparable BLR values as received by the use of the NWM data with a horizontal resolution of $0.125{^\circ } \times 0.125{^\circ }$. Thus, by using the lower resolved NWM data with $1{^\circ } \times 1{^\circ }$ the amount of data that needs to be processed during the ray-tracing can be reduced without an impact on the BLR.

4 The exclusion of unreliable sessions according to the criteria mentioned in Sect. 3.1.1 is done in a later step.

5 With respect to the analysis of the data set of the Continuous VLBI Campaign 2011 (CONT11; see Eriksson et al. 2014).

6 With respect to the analysis of the data set containing specific VLBI sessions in the interval 2011.0–2013.5 (see Eriksson et al. 2014).

7 The solutions of the parameterizations 1 and 3 have been compared. See Sect. 3.2 for the details on these analysis parameterizations.

8 Global Modeling and Assimilation Office Goddard Earth Observing System Model version 5.9.1 Forward Processing for Instrument Teams
